# Intentional Weight Loss and Dose Reductions of Anti-Diabetic Medications – A Retrospective Cohort Study

**DOI:** 10.1371/journal.pone.0032395

**Published:** 2012-02-27

**Authors:** Anita Ashok Kumar, Ghanshyam Palamaner Subash Shantha, Scott Kahan, Rohit Joshua Samson, Nelson David Boddu, Lawrence Jay Cheskin

**Affiliations:** 1 Department of Epidemiology, Johns Hopkins Bloomberg School of Public Health, Baltimore, Maryland, United States of America; 2 Department of Health Policy and Management, Johns Hopkins Bloomberg School of Public Health, Baltimore, Maryland, United States of America; 3 Department of Health Policy, George Washington University School of Public Health and Health Services, Washington, D. C., United States of America; 4 Johns Hopkins Weight Management Center, Johns Hopkins University, Baltimore, Maryland, United States of America; 5 Department of Health, Behavior and Society, Johns Hopkins Bloomberg School of Public Health, Baltimore, Maryland, United States of America; University of Las Palmas de Gran Canaria, Spain

## Abstract

**Background and Aim:**

Intentional weight loss, primarily by improving insulin resistance, is known to decrease the need for anti-diabetic medications. In this study, we assess the magnitude of weight loss that resulted in dose reductions or discontinuation of anti-diabetic medications in overweight or obese patients with type 2 diabetes (DM) undergoing weight loss treatment.

**Methods:**

Case records of 50 overweight or obese patients with DM who successfully decreased dosage or discontinued diabetes medications after losing weight via attendance at two University-based, outpatient weight management centers were analyzed. Follow-up visits, weight reduction interventions, and decisions for dose reductions or discontinuation of medications were individualized to patient needs by the treating physician.

**Results:**

Mean starting BMI was 35 kg/m^2^, mean age 53.4 years, and 58% were male. All 50 used at least one anti-diabetic medication (30 metformin, 39 sulfonylureas, 31 insulin, 21 sitagliptin) to manage blood sugar. Mean duration of follow-up was 30.2 months. Mean weight loss was 10.8±4.1 kgs (11.1% of initial body weight ±4.7%). 22/50 patients (44%) discontinued anti-diabetes medications (14 sulfonylureas [36%], 7 insulin [23%], 4 sitagliptin [19%]). The mean percentage weight loss achieved at the point of successful discontinuation of medication was 11.2%±3.5% (14% for sulphonylureas, 11% for insulin, and 7.1% for sitagliptin). Mean percentage weight loss of 5.6%±2.8% (5.1% for sulphonylureas, 4.3% for insulin, and 7.1% for sitagliptin) was required for initial dose reduction. For every 5% weight loss, predicted dose reductions were sulphonylureas, 39%; insulin, 42%; and any anti-diabetic medications, 49%.

**Conclusion:**

Among overweight or obese patients with type 2 diabetes, intentional weight loss of 7–14% was typically required for full discontinuation of at least one anti-diabetic medication. Discontinuation of insulin was achieved at a mean weight reduction of 11% of initial body weight.

## Introduction

National survey data for 2007–2008 indicate that 33.8% of US adults are obese [body mass index (BMI; calculated as weight in kilograms divided by height in meters squared)≥30], and the combined prevalence of overweight and obesity is reported to be 68% [1]. Obesity and overweight are associated with numerous medical problems such as hypertension, type 2 diabetes, coronary artery disease, dyslipidemia, gallbladder disease, sleep apnea, osteoarthritis, and hyperuricemia [2], [3].

Type 2 diabetes mellitus (DM) in obese patients is a particularly important condition. Data from the Behavioral Risk Factor Surveillance System showed that overweight (BMI 25–29.9), obese (BMI 30–39.9), and morbidly obese (BMI≥40) US adults had 1.59, 3.44 and 7.37 times higher odds, respectively, of diagnosed DM, compared to those with normal BMI (BMI 18.5–25) [3]. Further, on average, obese individuals with DM have a higher prevalence of end-organ damage and are at higher risk for cause-specific mortality compared to obese individuals without DM [4], [5]. Obesity and DM in unison also generate immense health care costs [6], [7].

DM in obese patients is predominantly related to insulin resistance; thus, intentional weight loss, by improving insulin resistance, decreases the risk of incident diabetes, and in patients with existing diabetes results in better glycemic control [8], [9]. The quantitative association between improvement in glycemia with weight loss and dose reductions or discontinuation of anti-diabetic medications is not well studied. This association would be clinically useful in treating, counseling and motivating patients in their weight loss efforts.

In this study of overweight and obese patients from two University-based weight management clinics, we studied the association between the percentage of weight loss and dose reductions or discontinuation of anti-diabetic medications.

## Materials and Methods

### Ethics statement

The study was approved by the Institutional Review Board of Johns Hopkins Bloomberg School of Public Health. Informed consent was not obtained from the study participants because the data was analyzed anonymously and was in accordance with Institutional Review Board guidelines. The Institutional Review Board verified the anonymity of data analysis performed in this study.

### Study setting and design

In this retrospective cohort study, case records of patients with BMI>25 kg/m^2^ at the time of enrollment into two specialty out-patient weight management centers, the Johns Hopkins Weight Management Center in Baltimore, MD and the George Washington Weight Management Program in Washington, DC were analyzed. Both were convenience samples. Patients were sampled if they enrolled in these centers during the period March 2008 to August 2010, had at least 6 months of follow-up data, reported taking anti-diabetic medications at the time of initial enrollment, and had at least one documented reduction in dosage or discontinuation of a prescribed anti-diabetic medication before the study concluded in March 2011. Exclusion criteria included: less than 6 months of follow-up, absence of dose reduction or discontinuation of anti-diabetic medication, no diagnosis of diabetes, prior surgical treatment of obesity, obesity associated with named syndromes, drug-induced obesity, endocrine causes of obesity, type 1 DM, pregnant and lactating women, and those with symptomatic coronary artery disease, chronic kidney disease, severe pulmonary artery hypertension, or physical limitations that prevented adherence to the prescribed physical activity.

### Baseline data collection

Case records of the study participants were reviewed, and demographic data (age, gender, race), cardiovascular risk factors (smoking, diabetes, hypertension), medication history (anti-diabetic medications, anti-hypertensive medications, anti-obesity medications, and lipid lowering drugs), clinical parameters (height, weight, systolic and diastolic blood pressure) and laboratory parameters {fasting glucose, HbA1C, total cholesterol, low density lipoprotein cholesterol (LDL), high density lipoprotein cholesterol (HDL), triglycerides} were collected. BMI was calculated as per standard guidelines [10]. Metabolic syndrome was determined using National Cholesterol Education program (NCEP ATP III) guidelines [11]. Type-2 diabetes was identified by physician diagnosis.

### Patient follow-up and weight management intervention details

Patients underwent team-based, comprehensive evaluation and treatment for weight loss. On an average, study participants had bimonthly clinic visits. Assessments consisted of physician-conducted medical history and physical examination, blood tests (as described above), and detailed dietary, behavioral, and exercise evaluations. Treatment was individualized, but consisted of a calorie-restricted diet, typically utilizing meal replacements, approximately 1000 kcal/day deficit, a behavior modification plan, and a plan for increasing physical activity utilizing both aerobic exercise and strength training. Follow-up visit frequency was variable, but typically involved weekly group support sessions and/or individual follow-up visits with a physician. Duration of treatment was also individualized, and in this sample ranged from 6 months to 3 years. The decision to alter the dose of or discontinue medications for weight-sensitive conditions was based upon the clinical judgment of the treating physician, taking into account relevant markers of control of the underlying condition (e.g., symptoms and signs, fingerstick and fasting blood glucose measurement, HbA1c). The biochemical and symptomatic response to medication regimen changes were monitored to ensure that modifications were neither premature nor unduly delayed.

### Outcome determination

The primary outcome of interest was discontinuation of any of the anti-diabetic medications. Other outcomes assessed were reduction in dose of anti-diabetic medications, mean and percentage weight loss at study exit, mean and percentage weight loss at the time of discontinuation of anti-diabetic medications, mean time to discontinuation of anti-diabetic medications, mean time to first dose reduction of anti-diabetic medications, mean and percentage weight loss at the time of first dose reduction of anti-diabetic medications, and average numbers of dose reductions for each of the anti-diabetic medications.

### Cohort summary

The study cohort was open with respect to entry and exit. The time origin for the study was entry or enrollment into the weight management program. The time metric was months of follow-up. Patients exited the cohort if they developed the primary outcome of interest or were administratively censored at study conclusion (in March 2011).

### Statistical analysis

Baseline characteristics of the study cohort and excluded patients were expressed in number (%) for categorical variables, and as mean ± standard deviation for continuous variables. Continuous and categorical variables were compared between the study cohort and the excluded patients using student's t test and chi-squared test as appropriate. The study cohort was further categorized into 3 BMI categories (overweight: BMI 25–29.99 kg/m^2^, obese: BMI: 30–39.99 kg/m^2^, morbidly obese: BMI≥40 kg/m^2^) at baseline as per standard guidelines [12]. Continuous and categorical variables were compared at baseline and at study conclusion between the three BMI categories using one-way analysis of variance and chi-square test, as appropriate. The changes in variables at study conclusion were compared with their baseline values for the entire cohort and within each of the three BMI categories using paired t-tests. At study conclusion, follow-up time was calculated in person-months for the total cohort and the three BMI categories. Missing data were imputed using multiple imputations with the previous observation carried forward [13]. Mean and percentage weight loss achieved at the end of one year, at each dose reduction, at each drug discontinuation, and at study conclusion were calculated for the entire cohort and individually for each of the three BMI categories. Simple and multiple linear regression analysis were performed to identify the unadjusted and adjusted association (adjusted for age, gender and ethnicity) between percentage weight loss (centered at 5%) and the mean percentage dose reductions of anti-diabetic medications (sulfonylureas, insulin, and all classes combined) for the total cohort. This analysis was not included for sitagliptin, metformin, or individually for the three BMI categories due to small numbers in these individual groups, which resulted in the regression model being unstable. Simple and multiple logistic regression analyses were performed to identify the unadjusted and adjusted association (adjusted for age, gender and ethnicity) between percentage weight loss (centered at 5%) and discontinuation of anti-diabetic medications (coded as categorical variables) for the total cohort. This analysis was performed for insulin, sulfonylureas and all classes combined, but not for sitagliptin, metformin and individual BMI categories, for the same reason described above. P value<0.05 was considered statistically significant. All analyses were performed using Stata 11.0 statistical software.

## Results

### Baseline characteristics

In total, 121 patient records (69 from Johns Hopkins and 52 from George Washington) were identified and reviewed. Of these, 50 patient records were excluded as they did not have a diagnosis of diabetes. The remaining 71 patients had a diagnosis of diabetes. Of these, 7 were excluded as they had less than 6 months follow-up data. Four more were excluded due to physical limitations that prevented adherence to the prescribed physical activity. 3 more were excluded because, though they were diabetic and successfully lost weight, they were unsuccessful in dose reductions of anti-diabetic medications. 7 more were excluded because, though diabetic, they neither lost weight nor were successful in reducing anti-diabetic medications. Hence, of the 71 diabetic patients, 21 were excluded and the remaining 50 patients formed the final study cohort.

In summary the final study cohort comprised of 50 patients (34 patients from Johns Hopkins and 16 from George Washington) with diabetes at the time of enrollment into the study who had at least one dose reduction or discontinuation of any of the anti-diabetic medications during the follow-up period. The proportion of smokers, patients with a diagnosis of hypertension, diabetes mellitus and metabolic syndrome were significantly higher in the study cohort when compared to the excluded patients (Table 1). Further, the study cohort had a higher mean fasting glucose and a higher mean HbA1c compared to the excluded patients (Table 1). However, the excluded patients had significantly higher mean LDL and total cholesterol compared to the study cohort (Table 1). With regard to medications, significantly higher proportions of participants in the study cohort took anti-diabetic, antihypertensive, anti-obesity, and lipid lowering drugs compared to excluded patients (Table 1).

**Table 1:** Baseline comparison between included patients (study cohort) and excluded patients.

Variables	Study cohort	Excluded patients	P-value
	(n = 50)	(n = 71)	
Age (yrs)	53.4±12.7	51.7±13.9	0.211
Males- n (%)	29 (58)	40 (56)	0.352
Caucasians – n (%)	33 (66)	43 (61)	0.106
African Americans – n (%)	17 (34)	28 (39)	0.113
*Current Smokers – n (%)	29 (58)	30 (42)	0.021
*Hypertension- n (%)	29 (58)	33 (46)	0.033
*Diabetes- n (%)	50 (100)	21 (30)	<0.001
*Metabolic syndrome–n (%)	26 (52)	30 (42)	0.031
Mean weight (Kgs)	136.5±24.0	127.0±38.1	0.126
Mean BMI (Kg/m^2^)	35.1±4.7	32.8±6.0	0.235
BMI categories- n (%)			
BMI ≤ 30	13 (26)	18 (25)	0.331
BMI: 31–39	30 (60)	44 (62)	0.173
BMI≥40	7 (14)	9 (13)	0.236
Systolic BP (mmhg)	143±13.2	148±23.6	0.116
Diastolic BP (mmhg)	91±9.37	89±11.8	0.237
*Fasting glucose (mg/dl)	111±58	92±21	0.011
*HbA1c %	8.6±1.6	6.9±1.1	0.021
*Total cholesterol (mg/dl)	205±46.4	252±23.7	0.039
*Total cholesterol (mmol/l)	5.30±1.2	6.51±0.6	0.039
*LDL (mg/dl)	147±59	163±36	0.016
*LDL (mmol/l)	3.8±1.5	4.2±0.9	0.016
HDL (mg/dl)	45±13	43±13	0.317
HDL (mmol/l)	1.2±0.3	1.1±0.3	0.317
Triglycerides (mg/dl)	174±47	169±54	0.184
Triglycerides (mmol/l)	1.98±0.50	1.92±0.61	0.184
*Anti-diabetes drugs- n (%)	50 (100)	21 (30)	<0.001
*Metformin- n (dose/day)	30 (1.75 g)	13 (1 g)	0.012
*Sulphonylureas- n	39	7	<0.001
*Glyburide- n (dose/day)	6 (10 mg)	2 (10 mg)	<0.001
*Glipiside- n (dose/day)	10 (10 mg)	2 (10 mg)	<0.001
*Glymipride- n (dose/day)	23 (2–4 mg)	5 (2–4 mg)	<0.001
*Sitagliptin- n (dose/day)	21 (100 mg)	1 (100 mg)	<0.001
*Insulin- n (dose/day)	31 (15 U)	2 (15 U)	<0.001
*Lipid Lowering drugs- n (%)	32 (64)	27 (38)	<0.001
*Anti-HTN drugs- n (%)	29 (58)	33 (46)	0.039
*Anti-obesity drugs- n (%)	8 (16)	9 (13)	0.051

*indicate significant differences (P-value<0.05).

LDL: Low Density Lipoprotein Cholesterol,

HDL: High Density Lipoprotein Cholesterol,

HTN: Hypertension,

BMI: Body Mass Index (weight in kilograms/height in meters squared).

Baseline characteristics of the study cohort (study cohort and the 3 BMI categories) are listed in Table 2 and Table S1. The mean age was 53.4±12.7 years. Males formed 58% of the cohort. 66% were Caucasian. Mean initial body weight was 136.5±24.0 kgs ; mean initial BMI was 35.1±4.7 kg/m^2^. When the three BMI categories were compared at baseline, the morbidly obese had significantly higher fasting glucose, total cholesterol, LDL cholesterol, HbA1C and serum triglycerides compared to the other two BMI categories (Table 2) (all P-values<0.05). Mean HDL was significantly lower in the morbidly obese compared to the other two BMI categories (Table 2). The proportion of patients with hypertension and metabolic syndrome was also significantly higher in the morbidly obese. The overweight and the obese did not differ significantly with regard to baseline variables.

**Table 2:** Baseline characteristics of the study patients according to BMI categories.

Variables	Entire cohort	BMI: 25–30	BMI: 31–39	BMI≥40	P values
	(n = 50)	(n = 13)	(n = 30)	(n = 7)	
Age (yrs)	53.4±12.7	52.5±13.6	53.1±16.4	53.4±14.9	0.136
Males- n (%)	29 (58)	5 (38)	19 (63)	5 (71)	0.355
Caucasians – n (%)	33 (66)	8 (62)	21 (70)	4 (57)	0.512
African Americans – n (%)	17 (34)	5 (38)	9 (30)	3 (43)	0.217
Current Smokers – n (%)	29 (58)	3 (23)	21 (70)	5 (71)	0.245
*Hypertension- n (%)	29 (58)	4 (31)	19 (63)	6 (86)	0.021
*Metabolic syndrome–n (%)	26 (52)	3 (23)	18 (60)	5 (71)	0.036
*Mean weight (Kgs)	136.5±24.0	122.1±18.6	133.4±16.8	142.4±18.6	0.013
*Mean BMI (Kg/m^2^)	35.1±4.7	28.9±0.7	35.2±3.3	43.7±2.4	0.025
Systolic BP (mmhg)	143±13.2	140±16.9	145±12.2	141±13.44	0.122
Diastolic BP (mmhg)	91±9.37	94±10.77	90±12.57	93±9.47	0.135
*Fasting glucose (mg/dl)	111±58	104±22	116±41	121±59	0.031
*HbA1c %	8.6±1.6	8.4±1.9	8.7±1.4	9.1±1.7	0.022
*Total cholesterol (mg/dl)	205±46.4	191±37.7	201±51.3	217±71	0.031
*Total cholesterol (mmol/l)	5.3±1.2	4.9±1.0	5.2±1.3	5.6±1.8	0.031
*LDL (mg/dl)	147±59	123±43.2	145±51.9	156±70.7	0.010
*LDL (mmol/l)	3.8±1.5	3.2±1.1	3.7±1.3	4.0±1.8	0.010
*HDL (mg/dl)	45±13	49±11	45±13.7	39±11.2	0.033
*HDL (mmol/l)	1.2±0.3	1.3±0.3	1.2±0.3	1.0±0.3	0.033
*Triglycerides (mg/dl)	174±47	156±52	182±67	188±32.8	0.028
*Triglycerides (mmol/l)	4.5±1.2	4.0±1.3	4.7±1.7	4.9±0.8	0.028
Anti-diabetes drugs- n (%)	50 (100)	13 (100)	30 (100)	7 (100)	0.175
Metformin- n (dose/day)	30 (1.75 g)	8 (1 g)	15 (1.75 g)	7 (2 g)	0.452
Sulphonylureas- n	39	7	21	11	0.611
Glyburide- n (dose/day)	6 (10 mg)	1 (10 mg)	2 (10 mg)	3 (10 mg)	0.365
Glipiside- n (dose/day)	10 (10 mg)	2 (10 mg)	2 (10 mg)	6 (10 mg)	0.514
Glymipride- n (dose/day)	23 (2–4 mg)	4 (2–4 mg)	17 (2–4 mg)	2 (2–4 mg)	0.156
Sitagliptin- n (dose/day)	21 (100 mg)	2 (100 mg)	15 (100 mg)	4 (100 mg)	0.356
*Insulin- n (dose/day)	31 (15 U)	3(10 U)	23 (15 U)	5 (20 U)	0.041
Lipid Lowering drugs- n (%)	32 (64)	5 (38)	21 (70)	6 (86)	0.128
Anti-HTN drugs- n (%)	29 (58)	4 (31)	18 (60)	7 (100)	0.138
Anti-obesity drugs- n (%)	8 (16)	0 (0)	5 (17)	3 (43)	0.112

*indicate significant differences (P-value<0.05). All are between group comparisons.

LDL: Low Density Lipoprotein Cholesterol,

HDL: High Density Lipoprotein Cholesterol,

HTN: Hypertension,

BMI: Body Mass Index (weight in kilograms/height in meters squared).

Other medication details at baseline are provided in supplemental file.

### Participant characteristics at study exit (Table 3)

**Table 3:** Participant characteristics at study exit.

Variables	Entire cohort	BMI:25–30	BMI: 31 to 39	BMI≥40	P values
	(n = 50)	(n = 13)	(n = 30)	(n = 7)	
Mean period of follow-up (months)	30.2±12.9	27.6±8.5	33.7±11	12±6.5	0.122
Person months of follow-up (months)	1740	403	1067	270	0.317
* Weight after one year (Kgs)	127.5±9.6	100.2±6.4	119.3±9.9	138.3±7.3	0.017
*Weight reduction after 1 year (Kgs)	12.7±2.1	13.6±3.6	11.3±2.4	7.3±3.1	0.029
*% weight loss after 1 year	14.2±3.2	15.1±4.6	13.9±3.8	9.2±3.3	0.011
* Weight at study exit (Kgs)	131.1±21.7	107±12.7	127±19.5	140.2±26.8	0.031
*Weight reduction at study exit (Kgs)	10.8±4.1	11.3±5.1	9.9±4.0	4.9±4.9	0.017
*% weight loss at study exit	11.1±4.7	12.8±3.9	11.7±5.1	7.2±4.4	0.013
*HbA1C (%) at study exit	8.1±1.2	7.6±1.1	8.0±0.6	8.9±0.3	0.037
*Reduction in HbA1C (%) at study exit	0.5±0.3	0.9±0.45	0.6±0.27	0.2±0.9	0.033
*Systolic blood pressure (mmhg)	138±19.1	136±14.7	140±15.2	147±21.3	0.012
*Diastolic blood pressure (mmhg)	86±8.5	82±9.9	87±10.1	90±8.45	0.017
Fasting glucose (mg/dl)	103±33	100±14	108±24	115±31	0.166
*Total cholesterol (mg/dl)	198±33.9	186±23.5	192±18.2	212±73	0.021
*Total Cholesterol (mmol/l)	5.1±0.8	4.8±0.6	4.9±0.4	5.5±1.9	0.021
*LDL (mg/dl)	143±28.9	116±27.2	132±26.8	151±47.3	0.017
*LDL (mmol/l)	3.6±0.7	3.0±0.7	3.4±0.7	3.9±1.2	0.017
*HDL (mg/dl)	44±11.8	49±13.1	46±11.2	37±7.1	0.026
*HDL (mmol/l)	1.1±0.3	1.3±0.3	1.2±0.3	0.9±0.2	0.026
* Triglycerides (mg/dl)	167±32.7	140±38	167±70	179±41.7	0.016
* Triglycerides (mmol/l)	4.3±0.8	3.6±1.0	4.3±1.8	4.6±1.2	0.016

*indicate significant differences (P-value<0.05), all are between group comparisons.

LDL: Low Density Lipoprotein Cholesterol,

HDL: High Density Lipoprotein Cholesterol,

BMI: Body Mass Index (weight in kilograms/height in meters squared).

Study exit: developed primary outcome of interest or administratively censored at study conclusion in March 2011.

On average, we had follow-up data for 30.2±12.9 months, and 1740 person-months of follow-up overall. Mean weight after 1 year of follow-up was 127.5±9.7 kgs, for a mean weight reduction of 12.7±2.1 kgs (14.2±3.2% of initial body weight). Mean weight at study exit was 131.1±21.7 kgs, for a mean weight reduction of 10.8±4.1 kgs (11.1±4.7% of initial body weight). The total cohort, the overweight, the obese and the morbidly obese had significantly (all P<0.05) lower weight, systolic blood pressure, diastolic blood pressure, fasting glucose, HbA1C, total cholesterol, LDL, and triglycerides compared to their baseline values (Tables 2 and 3). The overweight achieved greater mean weight loss, mean fasting glucose, and mean HbA1C reduction when compared to other BMI categories at study exit (Table 3). The morbidly obese had a higher mean systolic blood pressure, diastolic blood pressure, total cholesterol, LDL cholesterol and triglycerides compared to the other two BMI categories at study exit (Table 3). Mean HDL was lowest among patients in the morbidly obese category at study exit.

#### Dose reduction or discontinuation of anti-diabetic medications and weight loss

(Tables 2 and 4): Of the 50 patients in our study (overweight, 13; obese, 30; morbidly obese,7), 30 used metformin (overweight, 8; obese, 15; morbidly obese, 7), 39 sulfonylureas (overweight, 9; obese, 24; morbidly obese, 7), 31 insulin (overweight, 3; obese, 23; morbidly obese, 5) and 21 used sitagliptin (overweight, 2; obese, 15; morbidly obese, 4). The average dose/day for these medications are detailed in Table 2. The three BMI categories did not differ with respect to average dose/day for these medications, except that morbidly obese patients were receiving a higher dose of insulin compared to the other two categories.

**Table 4:** Weight loss and details of anti-diabetes drug dose reduction/discontinuation.

Variables	Entire cohort	BMI:25–30	BMI: 31–40	BMI≥41
	(n = 50)	(n = 13)	(n = 30)	(n = 7)
No. using anti-diabetic medications initially	50	13	30	7
n (%)¶ discontinued anti-DM medication	22 (44)	5 (38)	14 (47)	3 (43)
% weight loss at any anti-DM discontinuation	11.2±3.5	8.9±2.4	11.3±3.1	14.1±2.1
n (%)¶ who had at least one dose reduction	50 (100)	13 (100)	30 (100)	7 (100)
% weight lost at first dose reduction	5.6±2.8	3.8±2.1	5.6±2.3	4.9±2.8
No. of dose reductions of any anti-DM	54	29	20	5
No. using Metformin at study initiation	30	8	15	7
No. who discontinued Metformin	0	0	0	0
No. with one dose reduction of Metformin	0	0	0	0
No. using SU at study initiation	39	7	21	11
n (%)¶ who discontinued SU	14 (36)	4 (57)	10 (48)	0 (0)
No. who discontinued glypiside	3	2	1	0
Time to discontinuing SU (months)	15.2±6.5	9.7±3.1	16.2±7.2	-
% weight lost at SU discontinuation	14±4.5	9±3.2	16±7.8	-
n (%)¶ with one dose reduction of SU	20 (51)	5 (71)	15 (71)	-
Time to first dose reduction of SU (months)	7.1±1.9	5.2±1.1	7.1±2.4	-
No. of dose reductions of SU	42	13	29	-
% weight loss at first dose reduction of SU	5.1±1.2	4.2±1.7	5.4±1.8	-
No. using Glyburide at study initiation	6	1	2	3
No. who discontinued glyburide	2	1	1	0
No. using Glymipride at study initiation	23	4	17	2
No. who discontinued glymipride	9	2	7	0
No using glypiside at study initiation	10	2	2	6
No. using Insulin at study initiation	31	3	23	5
n (%)¶ who discontinued insulin	7 (23)	2 (67)	4 (17)	-
% weight lost at Insulin discontinuation	11.4±1.5	10.1±1.8	11.3±1.2	-
Time to discontinuing insulin (months)	14.5±6.8	11.1±3.8	15.8±8.1	-
n (%)¶ with at least one insulin dose reduction	15 (48)	3 (100)	14 (61)	2 (40)
Time to first dose reduction of Insulin	4.5±1.2	3.8±0.9	4.6±1.6	6.1
% weight loss at first dose reduction of Insulin	4.3±1.5	3.1±1.3	4.7±1.1	5.1
No. of dose reductions of Insulin	24	16	39	4
No. using SGT at study initiation	21	2	15	4
n (%)¶ who quit SGT	4 (19)	1 (50)	2 (13)	1 (25)
% weight lost at SGT discontinuation	7.1±2.1	7.2±1.9	6.8±2.3	7.5
Time to discontinuing SGT	16±3.1	16.1±2.5	15.5±2.1	17
n(%)¶ with at least one dose reduction of SGT	4 (19)	1 (50)	2 (13)	1 (25)
Time to first dose reduction of SGT	16±3.1	16.1±2.5	15.5±2.1	17
% weight loss at first dose reduction of SGT	7.1±2.1	7.2±1.9	6.8±2.3	7.5
No. of dose reductions of SGT	1	1	1	1
No. who quit both Insulin and SU	3	1	2	0

Anti-DM: anti-diabetes medications.

SU: Sulphonylureas, SGT: Sitagliptin,

BMI: Body Mass Index (weight in kilograms/height in meters squared).

¶ : % mentioned are with respect to the total number in each group who were using that particular drug at study initiation.

By study exit, 44% (22/50) of the study patients were successful in discontinuing one or more of their anti-diabetic medications. Mean weight loss associated with the discontinuation of one or more of these medications was 15.3±5.8 kgs. However, 1 patient was able to discontinue one of his anti-diabetic medications (insulin) at a weight loss of just 3.6 kgs and one patient needed to lose 28.6 kgs to discontinue one of his anti-diabetic medications (insulin). Mean percentage weight loss associated with the discontinuation of one or more of these medications was 11.2±3.5%.

Mean weight loss associated with at least one dose reduction was 7.7±3.8 kgs. At the extremes, one patient was successful in having a dose reduction of his insulin after a weight loss of just 2.3 kgs, while one patient had to lose 17.7 kgs to achieve an insulin dose reduction. A mean 5.6±2.8% weight loss was associated with at least one dose reduction.

When individual anti-diabetic medication classes were analyzed, none of the patients discontinued or reduced the dose of metformin, but 36% (14/39) of patients using sulfonylureas discontinued this medication, 23% (7/31) of those using insulin discontinued this medication, and 19% (4/21) of those using sitagliptin discontinued this medication. The mean percentage weight loss that was associated with discontinuing sulfonylureas, insulin and sitagliptin were 14.0% (19.1±8.2 kgs), 11.4% (15.6±5.5 kgs), and 7.1% (9.7±2.1 kgs), respectively.

With respect to dose reductions, 51% (20/39) of those using sulfonylureas, 48% (15/31) of those using insulin and 19% (4/21) of those using sitagliptin achieved at least one dose reduction of the respective medications. The mean percentage weight loss that was associated with at least one dose reduction of sulfonylureas, insulin, and sitagliptin were 5.1% (7.0±1.6 kgs), 4.3% (5.9±1.9 kgs) and 7.1% (9.7±2.1 kgs), respectively.

### Association between percentage weight lost and predicted percentage dose reduction of anti-diabetic medications

For every 5% weight loss, on average, the dose of sulfonylureas, insulin and any of the anti-diabetic medications was predicted to be reduced by 39%, 42% and 49%, respectively (Table 5). Also, for every 5% reduction in weight, the odds ratio of discontinuing sulfonylureas, insulin and any of the anti-diabetic medications were 1.24, 1.30 and 1.37, respectively (Table 6).

**Table 5:** Linear regression analysis associating percentage weight loss (every 5%) and mean % dose reductions of anti-diabetes medications (entire cohort).

Anti-diabetes medications	Mean % dose reductions		
	Mean %	95% C.I	P-value
Sulphonylureas			
Unadjusted	45	13–65	0.023
*Adjusted	39	18–71	0.039
Insulin			
Unadjusted	51	11–69	0.041
*Adjusted	42	14–64	0.044
Any anti-DM medications			
Unadjusted	58	18–73	0.027
*Adjusted	49	31–70	0.031

*adjusted for age, gender and ethnicity.

Anti-DM: antidiabetic.

**Table 6:** Logistic regression analysis associating percentage weight loss (every 5%) and discontinuation of anti-diabetes medications (entire cohort).

Anti-diabetes medications	Discontinuation of anti-diabetes medications		
	Odds ratio	95% C.I	P-value
Sulphonylureas			
Unadjusted	1.31	1.04–1.55	0.041
*Adjusted	1.24	1.01–1.49	0.045
Insulin			
Unadjusted	1.39	1.07–1.63	0.039
*Adjusted	1.30	1.04–1.57	0.042
Any anti-DM medications			
Unadjusted	1.49	1.08–1.70	0.033
*Adjusted	1.37	1.12–1.72	0.042

*adjusted for age, gender and ethnicity.

Anti-DM: antidiabetic.

## Discussion

It is well-demonstrated that lifestyle changes, such as decreased caloric intake and increased physical activity leading to weight loss can decrease the incidence and progression of DM [9], [14], [15]. In this study, we examined the magnitude of weight loss associated with the reduction of dose or discontinuation of anti-diabetic medications in a selected sample of patients with DM who attended 2 University-based weight management centers. While a number of previous studies suggest that weight loss often leads to diminished need for anti-diabetic medications [16], [17] we are not aware of any previous study that aimed to quantify the magnitude of weight loss associated with an improvement in blood sugars sufficient to warrant a decrease in dose, or discontinuation of anti-diabetes medications. Though trials like Look AHEAD have analyzed the economic impact of anti-diabetic drug dose reductions consequent to weight loss [16], our observational study appears to be the first to identify the association between the magnitude of the weight loss and dose reductions or discontinuations of anti-diabetic medications.

This retrospective cohort study found that the selected patients achieved 14.2% mean weight loss at one year, and 11.1% at study conclusion (30.2 months mean follow-up), respectively (Table 2). The high magnitude of weight loss is not necessarily surprising given that this sample was selected to identify those patients who were successful enough in their lifestyle changes to be able to lower the dose or discontinue at least one anti-diabetic medication. Further, by virtue of being enrolled in a University-based, comprehensive weight management center program, they each had access to an intensive, multi-specialist support team that utilized state-of-the-art weight management tools and techniques. 44% of these patients with type 2 diabetes successfully discontinued one or more of their anti-diabetes medications. On average, 11.2% of initial body weight loss was required to achieve this (Table 3).

We observed that the percentage weight loss that was associated with discontinuing or reducing the dose of insulin was lower than that for sulfonylureas. The fact that insulin is shorter acting than sulfonylureas, and that insulin involves injections, may have prompted the treating physicians to discontinue insulin earlier than sulfonylureas. Also, we observed that none of the patients in our cohort discontinued metformin or had even a single dose reduction. The reason for this is likely that the treating physicians preferred retaining metformin for its weight-reducing and insulin resistance-decreasing properties, rather than for its effect on glycemia [18]. In additional, because there is minimal risk of hypoglycemic effects with metformin, compared with insulin or sulfonylureas, less aggressive discontinuation of metformin may be preferred.

Also, it is encouraging to note that 3 of the 4 different classes of anti-diabetic medications used in our study could be dose reduced/discontinued with weight loss. Hence it should be emphasized that, irrespective of the type of anti-diabetic medication and its mechanism of action, weight loss indeed helps in dose reduction/discontinuation and hence should be uniformly prescribed to overweight and obese patients with type-2 diabetes mellitus.

Our observation that 50 patients (study cohort) who could lose weight were also successful in dose reductions/discontinuation of anti-diabetic medications while only 3 who lost weight were unsuccessful in dose reductions/discontinuation of anti-diabetic medications is encouraging.

Our study participants in the morbidly obese category showed some interesting differences. They tended to have poorer cardiovascular risk profiles at baseline, and achieved lesser reduction of body weight, blood pressure, and lipid levels (Tables 1, 2 and 3). Also, while some had dose reductions, none was successful in discontinuing any of their anti-diabetes medications. The possible reason for this observation could be that the participants in the morbidly obese category had a higher HbA1c% at baseline (Table 2). Though they did achieve significant reduction in their HbA1c% at study exit (Table 3) this may not have been sufficient to translate into discontinuation of any of the anti-diabetic medications. Hence, it may be reasonable to hypothesize that greater magnitudes of weight loss may be required for the participants in this group to discontinue anti-diabetic medications when compared to the participants in the other BMI categories.

Evidence from observational studies and clinical trials has clearly shown that morbidly obese patients with type 2 DM benefit from bariatric surgery [19], [20], [21]. Nearly 50–80% of these patients achieved complete remission of diabetes. When followed, prevalence of diabetes decreased at 2 years, 8 years and even after 10 years after surgical intervention in these patients [20]. Further, bariatric surgery provided sustained improvement in quality of life and significantly reduced mortality [21]. At the end of 2 years, in a clinical trial setting, surgically treated patients had lost 20% of their body weight and reduced their HbA1c% by 2.5 [20]. However, in our study, morbidly obese patients lost a lesser amount, 7.2% of their body weight, and reduced HbA1c% by 0.2 by study exit (Table 3). In addition to reduction in insulin resistance, bariatric surgery is known to increase incretins like glucagon-like peptide-1 (GLP-1) and glucose-dependent insulinotropic polypeptide (GIP), which lead to improved beta-cell function [21]. This may help explain the dramatic and sustained response that bariatric surgery can achieve in the morbidly obese when compared to average results using medical management. Diabetes management has moved from a guidelines-based approach to an individualized approach that takes factors such as age, comorbidities, diabetes-associated complications, symptoms and patient satisfaction into account in deciding on treatment choices and doses [22]. In a recent review by Ismail-Beigi et al., the authors described the superiority of individualized diabetes management over guideline-based diabetes management after pooling multiple trial data [22]. Consistent with this novel approach, our study, which was based on individualized and comprehensive management of obese diabetic patients, took the overall clinical profile of the patient, rather than any single factor into making treatment choices. Hence, the results of our study may be applicable to any physician-supervised, outpatient weight management approach. It is encouraging that success at decreasing and discontinuing anti-diabetic medications was achieved with relatively modest weight loss, and that even a medication with a very substantial impact on patient health-related quality of life - insulin [23] - was discontinued at levels of weight loss that can often be achieved in physician-guided settings.

Small sample size is an important limitation of our study that decreased the precision of our estimates and prevented us from examining effect modification attributable to gender, race and BMI categories. The retrospective cohort design limits our data to whatever is recorded in patient case records. The convenience sampling strategy, including only those obese patients with diabetes who successfully achieved at least one dose reduction of their anti-diabetic medications may have introduced a selection bias, by selecting individuals who were well motivated or consistent in their attendance at follow-up visits, and thus may not be representative of all clinic attendees, or all patients with type-2 diabetes. Missing data was another limitation. Though we used statistical adjustments, it is still possible that the estimates might have been biased due to these missing data. Also, it would have been interesting to compare our study cohort with patients who had significant weight loss but were unsuccessful in dose reductions/discontinuation of anti-diabetic medications. We were unable to do this analysis as we had only 3 patients in this category.

In conclusion, among the obese patients with type 2 diabetes studied, intentional weight loss of a mean magnitude of 7–14% was typically required for full discontinuation of at least one anti-diabetic medication. Discontinuation of insulin was achieved at a mean weight reduction of 11% of starting body weight. Successful reduction in dosage of anti-diabetes medications was typically achieved with a lesser, 4–7% weight loss. Also, with just 5% weight loss, doses of sulphonylureas, insulin and any of the anti-diabetic medications could be reduced by 39%, 42% and 49% respectively. Knowledge of the modest magnitude of weight loss typically required to successfully reduce medication use among overweight and obese patients with type 2 diabetes may be helpful to health care providers and patients alike.

## Supporting Information

Table S1 Medication details of the study participants at baseline.
(DOCX)
