# Supplementary material for: Intentional Weight Loss and Dose Reductions of Anti-Diabetic Medications – A Retrospective Cohort Study
Source: PLoS One. 2012 Feb 27;7(2):e32395. doi: 10.1371/journal.pone.0032395 (PMC3288092; doi:10.1371/journal.pone.0032395)
Supplement: Table S1 — Medication details of the study participants at baseline. (DOCX) [file pone.0032395.s001.docx]

**Table S1: Medication details of the study participants at baseline**

| Variables | Entire cohort | BMI ≤ 30 kg/m^2^ | BMI: 31–39 kg/m^2^ | BMI ≥ 40 kg/m^2^ |
| --- | --- | --- | --- | --- |
|  | (n = 50) | (n = 13) | (n = 30) | (n = 7) |
| Lipid Lowering drugs- n (%) | 32 (64) | 5 (38) | 21 (70) | 6 (86) |
| Atorvastatin- n (dose/day) | 20 (20mg) | 4 (20mg) | 10 (20mg) | 6 (20mg) |
| Rosuvastatin- n (dose/day) | 7 (20mg) | 1 (20mg) | 3 (20mg) | 3 (20mg) |
| Finofibrate- n (dose/day) | 5 (400mg) | 1 (400mg) | 1 (400mg) | 3 (400mg) |
| Anti-HTN drugs- n (%) | 29 (58) | 4 (31) | 18 (60) | 7 (100) |
| Hydrocholorothiazide- n (dose/day) | 14 (25mg) | 2 (25mg) | 9 (25mg) | 3 (25mg) |
| Atenolol- n (dose/day) | 19 (50mg) | 2 (50mg) | 15 (50mg) | 2 (50mg) |
| ACE inhibitors- n (dose/day) | 27 (10mg) | 4 (10mg) | 20 (15mg) | 3 (20mg) |
| ARBs- n (dose/day) | 11 (50mg | 1 (50mg) | 5 (50mg) | 5 (50mg) |
| Anti-obesity medications- n (%) | 8 (16) | 0 (0) | 5 (17) | 3 (43) |
| Rimonaband- n (dose/day) | 3 (6) | 0 (0) | 1 (3) | 2 (29) |
| Orlistat- n (dose/day) | 5 | 0 (0) | 3 (10) | 2 (29) |
